# Supplementary figures and images for: YAP/TAZ inhibition refines TGF-β signaling to prevent laryngeal fibrosis
Source: bioRxiv. 2026 May 21:2026.05.18.726086. Preprint. [Version 1] doi: 10.64898/2026.05.18.726086 (PMC13228622; doi:10.64898/2026.05.18.726086)

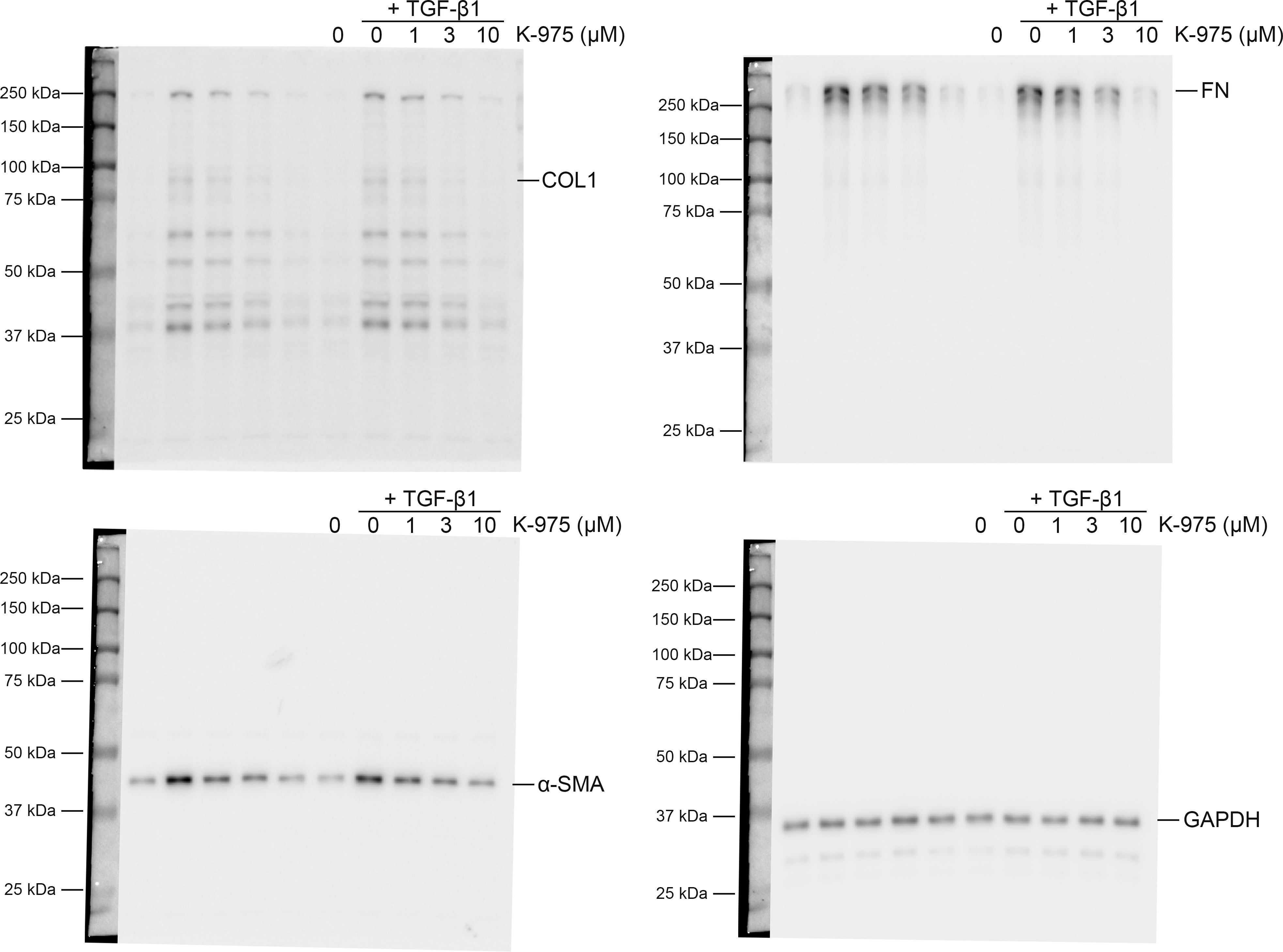

Supplement: Supplement 2 [file media-2.jpg]

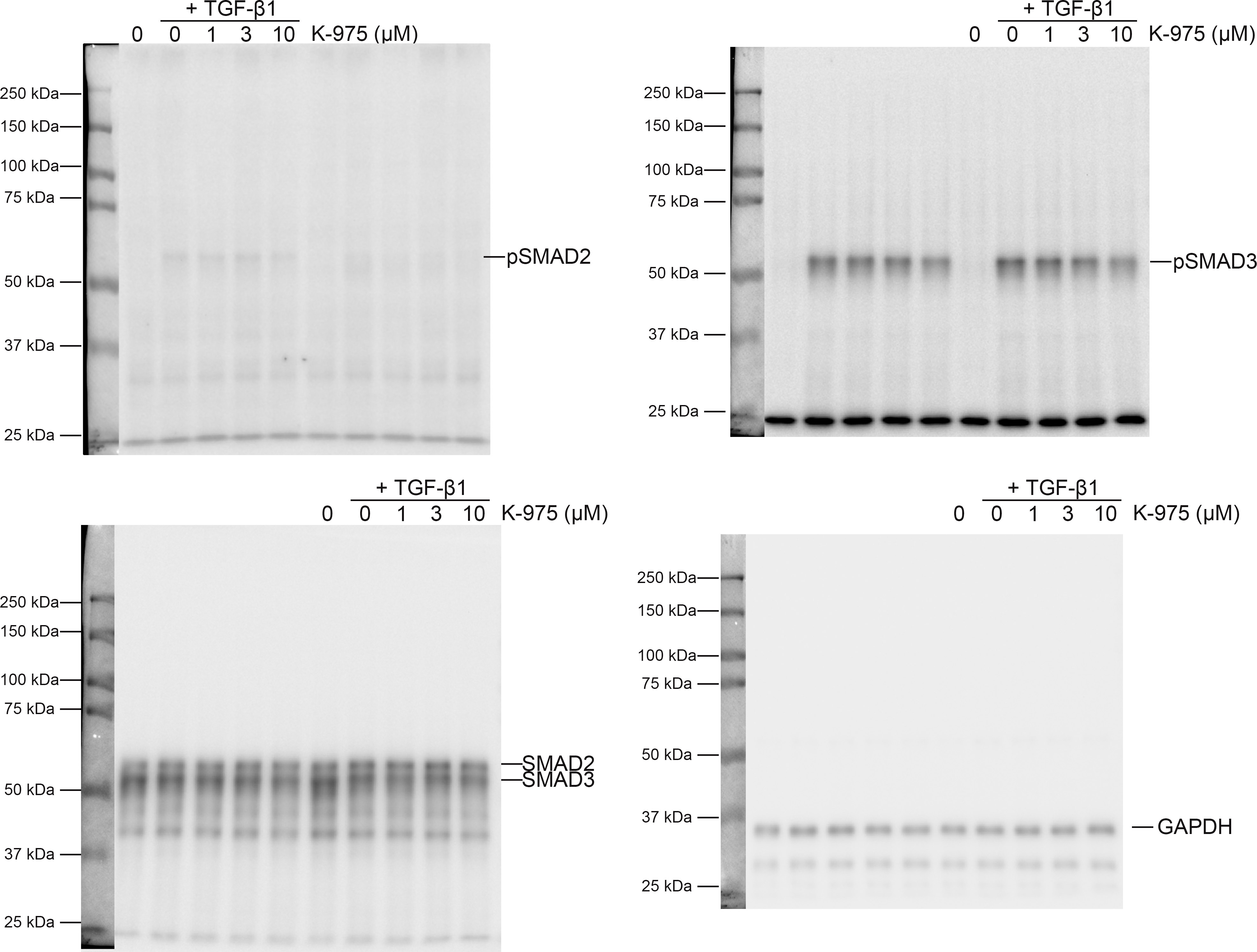

Supplement: Supplement 3 [file media-3.jpg]
